# Salivary kynurenine pathway metabolites as potential non-invasive markers of glandular dysfunction in Sjögren’s disease

**DOI:** 10.1038/s41598-025-24287-y

**Published:** 2025-11-18

**Authors:** Youngjae Park, Young-Seok Song, Jung Hee Koh, Jennifer Jooha Lee, Wan‑Uk Kim, Sung-Hwan Park, Seung-Ki Kwok

**Affiliations:** 1 Division of Rheumatology, Department of Internal Medicine, Seoul St. Mary’s Hospital, College of Medicine, The Catholic University of Korea, Seoul, Republic of Korea; 2 Department of Biomedicine & Health Sciences, College of Medicine, The Catholic University of Korea, Seoul, Republic of Korea; 3 Division of Rheumatology, Department of Internal Medicine, Uijeongbu St. Mary’s Hospital, College of Medicine, The Catholic University of Korea, Seoul, Republic of Korea

**Keywords:** Sjögren’s disease, Salivary biomarker, Kynurenine metabolite, Salivary gland dysfunction, Quinolinic acid, Biomarkers, Medical research, Rheumatology

## Abstract

Sjögren’s disease (SjD) is an autoimmune disorder characterized by salivary gland dysfunction and systemic manifestations. This study aimed to evaluate kynurenine (Kyn) pathway metabolites in saliva and investigate their clinical relevance in SjD. Saliva samples were collected from 39 SjD patients and 32 healthy controls (HCs). Concentrations of tryptophan, Kyn, quinolinic acid (QA), and kynurenic acid (KA), as well as inferred enzyme activities, were measured using ELISA. Receiver operating characteristic curve analyses were conducted, and correlations with disease activity indices and unstimulated whole salivary flow rates (UWSFR), were assessed. SjD patients exhibited significantly higher salivary KA levels (*p* < 0.001) and lower QA levels (*p* < 0.0001) compared with HCs. Ratios of QA to Kyn and KA to Kyn were also significantly altered in the SjD group. Salivary QA demonstrated excellent discriminatory ability (area under the curve > 0.85, sensitivity 0.87, specificity 0.76, *p* < 0.001) for distinguishing SjD from HCs. Among various clinical parameters, salivary QA levels showed a strong inverse correlation with UWSFR (*r* = − 0.596, *p* < 0.001). Salivary Kyn pathway metabolites, particularly QA, may serve as non-invasive biomarkers reflecting salivary gland dysfunction in SjD.

**Supplementary Information:**

The online version contains supplementary material available at 10.1038/s41598-025-24287-y.

## Introduction

Sjögren’s disease (SjD) is a chronic systemic autoimmune disease predominantly characterized by lymphocytic infiltration and destruction of exocrine glands, leading to dry mouth and dry eyes^1^. However, SjD extends beyond glandular dysfunction, encompassing systemic manifestations, constitutional symptoms, and a markedly impaired quality of life^2^. Despite advances in understanding the immunopathology of SjD, including its complex interplay between epithelial cells, B cells, T cells, and cytokines such as interferon-gamma (IFN-γ), current diagnostic tools still rely heavily on invasive procedures such as salivary gland biopsy, and serologic markers that may not accurately reflect glandular involvement or disease activity^1–3^.

In recent years, increasing attention has been paid to metabolic reprogramming in autoimmune diseases. Immune cell activation and chronic inflammation are accompanied by changes in bioenergetics and amino acid metabolism^4^. One such pathway, the kynurenine pathway (KP) of tryptophan degradation, is particularly relevant due to its immunomodulatory properties^5^. Tryptophan is metabolized into kynurenine (Kyn) by indoleamine 2,3-dioxygenase 1 (IDO1), which is upregulated by inflammatory stimuli such as IFN-γ^6^. Downstream metabolites of this pathway, including kynurenic acid (KA) and quinolinic acid (QA), are generated by enzymes such as kynurenine aminotransferases (KAT) and kynurenine-3-monooxygenase (KMO), respectively^6^. These metabolites influence immune responses by modulating T cell function, oxidative stress, and neurotoxicity^5–7^.

Previous studies have demonstrated altered KP metabolite profiles in systemic lupus erythematosus (SLE), rheumatoid arthritis (RA), and more recently, in SjD^8–12^. For example, Kyn and Kyn/tryptophan ratios in serum have been linked to disease activity and fatigue in SjD patients^11^. Additionally, Urbanski et al. identified Kyn as a key component of a metabolic signature predictive of SjD using labial salivary gland tissue^13^. However, few studies have investigated KP metabolites directly in saliva, which represents a non-invasive and potentially more gland-specific sample matrix.

Given the critical role of salivary gland epithelial cells in the initiation and propagation of local immune responses in SjD^3^, salivary analysis may offer a more accurate reflection of glandular immunometabolic activity. Moreover, the use of salivary biomarkers aligns with the clinical need for less invasive, more accessible diagnostic tools. A recent study by Florezi et al. using targeted metabolomics confirmed that saliva mirrors metabolic changes in the glandular microenvironment and suggested that it may be suitable for detecting oxidative stress and immune activation in SjD^14^.

In this study, we sought to investigate the levels of tryptophan and its KP metabolites such as Kyn, KA, and QA in the saliva of patients with primary SjD and healthy controls (HCs). We further aimed to evaluate the activities of enzymes involved in this pathway by calculating metabolite ratios (Kyn/Trp for IDO1, KA/Kyn for KAT, and QA/Kyn for KMO), and to examine correlations between these metabolites and clinical manifestations and glandular function. Our goal was to assess the clinical utility of salivary KP metabolites as biomarkers in SjD.

## Methods

### Population

All subjects included in this study were selected from participants in the Korean Initiative of Primary Sjögren’s Syndrome (KISS) cohort. Selection was based on (1) the availability of stored unstimulated saliva samples obtained at the time of cohort enrollment and (2) fulfillment of the 2016 American College of Rheumatology/ European League Against Rheumatism (EULAR) classification criteria for primary SjD^15^. The KISS cohort is a nationwide, prospective database containing clinical information and biological samples from patients with primary SjD in Korea. Detailed information regarding the cohort has been described elsewhere^16^. HCs were recruited from community volunteers without autoimmune or chronic inflammatory diseases. Although the HC group was not strictly age- and sex-matched to the SjD group, the overall distributions were comparable, as summarized in Table 1. Informed consent was obtained from all participants in accordance with the principles of the Declaration of Helsinki. All studies related to this cohort were approved by the Institutional Review Board of Seoul St. Mary’s Hospital, The Catholic University of Korea (approval number: KC13ONMI0646).

**Table 1:** Demographic information of patients with Sjögren’s disease and healthy controls.

	SjD, *n* = 39	HCs, *n* = 32
Age at baseline, years	54 (47–61)	52 (42–61)
Age at diagnosis, years	47 (38–58)	
Sex, female	38 (97.4)	32 (93.8)
Disease duration, months	6 (0–36)	
Dry eye	39 (100)	
Dry mouth	37 (94.9)	
UWSFR ≤ 0.1 ml/min	25 (64.1)	
Focus score	3 (2–8)	
ESSDAI total score	4 (0–7)	
ESSPRI overall score	4.7 (4.0–6.8)	
Any extraglandular manifestations	26 (66.7)	
Pilocarpine	33 (84.6)	
Corticosteroid	19 (48.7)	
Hydroxychloroquine	20 (51.3)	
Anti-Ro positivity	33 (84.6)	
Anti-La positivity	19 (48.7)	
Rheumatoid factor positivity	25 (64.1)	
Low C3	8 (20.5)	
Low C4	4 (10.3)	
Hypergammaglobulinemia	9 (23.1)	

All data are expressed as number (%) or median (interquartile range). SjD: Sjögren’s disease, HCs: healthy controls, UWSFR: unstimulated whole salivary flow rate, ESSDAI: EULAR Sjogren’s syndrome disease activity index, ESSPRI: EULAR Sjogren’s syndrome patient-reported index.

### Clinical parameters

Clinical information used in the present study was extracted from the KISS cohort database. The analyses included demographic data, secretory function of the salivary glands measured by unstimulated whole salivary flow rate (UWSFR), and serological status such as the presence of autoantibodies. Systemic disease activity and disease-related symptom severity were assessed using the EULAR Sjögren’s Syndrome Disease Activity Index (ESSDA; range, 0–123)^17^ and the EULAR Sjögren’s Syndrome Patient-Reported Index (ESSPRI; range, 0–10)^18^. The presence of extraglandular manifestations (EGMs) was defined as the presence of any items listed in the Supplementary Table 1.

### Measurement of salivary biomarkers

Saliva samples were collected at the same time points as the clinical assessments. Unstimulated whole saliva was collected at the time of UWSFR measurement. Participants fasted for at least 3 h before sampling, and early morning collection after an overnight fast of ≥ 8 h was recommended. Collected saliva was processed within 1 h. Samples were centrifuged at 8000 rpm for 1 min at 4 °C to remove debris and cells, and the resulting supernatants were immediately stored at -80 °C until analysis. No additional protease inhibitors were used. The concentrations of IFN-γ, tryptophan, IDO1, Kyn, KA, and QA in the saliva of patients with SjD and HCs were measured using enzyme-linked immunosorbent assay kits (IFN-γ: R&D Systems, Minneapolis, MN, USA; tryptophan, IDO1, Kyn, KA and QA: MyBioSource, San Diego, CA, USA). Enzymatic activities related to the KP were assessed by calculating the following ratios: Kyn to tryptophan (IDO1 activity), KA to Kyn (KAT activity), and QA to Kyn (KMO activity)^19^.

### Statistical analysis

Continuous variables are presented as medians with interquartile ranges, and categorical variables are presented as numbers and percentages. Differences in biomarker levels between study groups were assessed using the Mann–Whitney U test. Correlations between biomarker levels and clinical parameters were evaluated using Spearman’s rank correlation coefficient. Statistical significance was defined as a *p* value < 0.05. All statistical analyses were conducted using IBM SPSS Statistics version 24.0 (SPSS Inc., Chicago, IL, USA), and graphical representations were generated with GraphPad Prism version 8.0 (GraphPad Software, San Diego, CA, USA).

## Results

### Clinical characteristics of the study population

A total of 39 patients with SjD and 32 HCs were included in the study (Table 1). Patients with SjD had a median age of 54 years and were predominantly female (97.4%). Common clinical features included xerostomia (94.9%), keratoconjunctivitis sicca (100%), and reduced UWSFR (64.1%). EGMs were present in 66.7% of patients. The median ESSDAI was 4, while the ESSPRI had a median of 4.7.

### Alterations in salivary kynurenine pathway metabolites

**Fig. 1:**
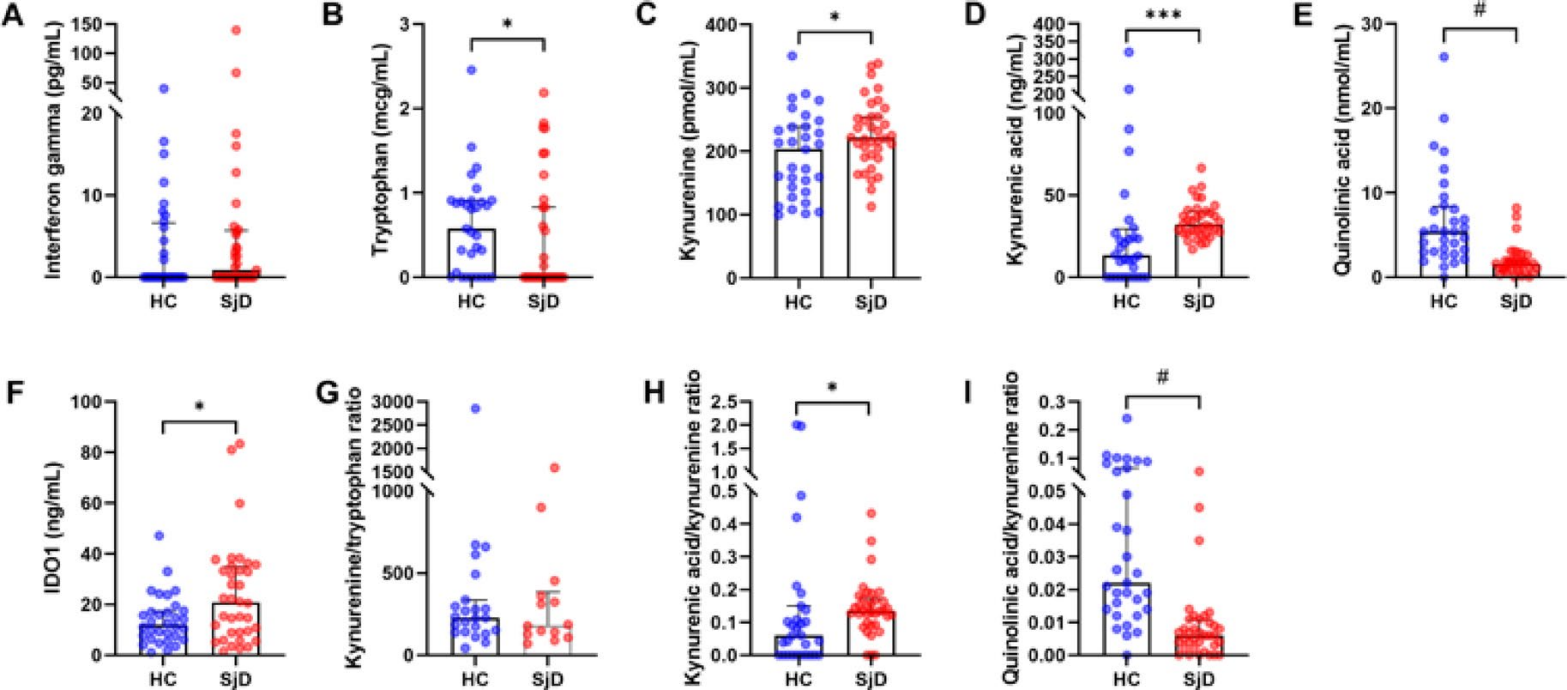
Tryptophan and kynurenine metabolites in saliva from HC and SjD patients. (**A**) Interferon gamma, (**B**) tryptophan, (**C**) kynurenine, (**D**) kynurenic acid, (**E**) quinolinic acid, (**F**) IDO1, (**G**) ratio of kynurenine to tryptophan, (**H**) ratio of kynurenic acid to kynurenine, and (**I**) ratio of quinolinic acid to kynurenine. All statistical analyses were performed using the Mann-Whitney U test. Bars indicate the median and interquartile ranges. HC: healthy controls, SjD: Sjogren’s disease, IDO1: indoleamine-2,3-dioxygenase 1, *p < 0.05, ***p < 0.001, #: *p* < 0.0001.

Salivary concentrations of key KP metabolites showed significant differences between SjD patients and HCs (Fig. 1). IFN-γ levels did not differ significantly between the two groups (Fig. 1A). Tryptophan levels were moderately reduced in SjD patients (Fig. 1B; *p* < 0.05), while Kyn levels were significantly elevated (Fig. 1C; *p* < 0.05). KA levels were significantly increased (Fig. 1D; *p* < 0.001), whereas QA levels were markedly decreased in SjD patients (Fig. 1E; *p* < 0.0001). Salivary IDO1 protein levels were higher in SjD patients (Fig. 1F; *p* < 0.05). However, the Kyn/Tryptophan ratio, representing IDO1 activity, showed no significant differences between the two groups (Fig. 1G). The KA/Kyn ratio (Fig. 1H) was significantly increased (*p* < 0.05), suggesting increased KAT activity, while QA/Kyn ratio (Fig. 1I) was markedly decreased in SjD patients (*p* < 0.0001), indicating reduced KMO activity. These findings suggest a distinct shift in KP metabolism in SjD compared to healthy individuals.

### Discriminatory performance of salivary metabolites

**Fig. 2:**
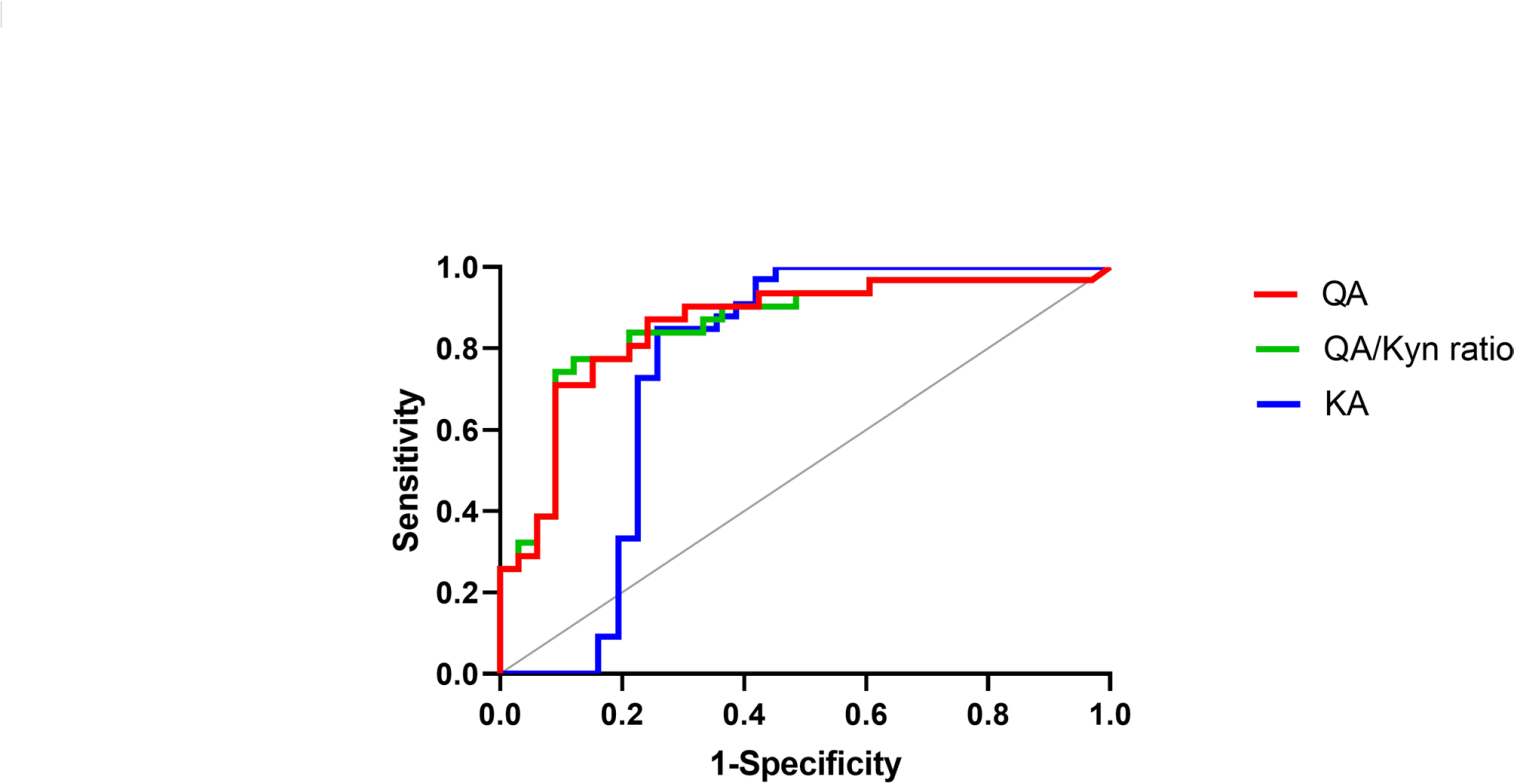
Receiver operating characteristic curves of salivary biomarkers for Sjögren’s disease. QA - red line, ratios of QA to Kyn - green line, KA - blue line. QA: quinolinic acid, Kyn: kynurenine, KA: kynurenic acid.

Next, we assessed discriminatory performance of salivary KP metabolites between SjD and HCs. Salivary metabolites that showed high statistical significance were included in analyses; KA, QA, and the QA/Kyn ratio. Receiver operating characteristic (ROC) curve analysis demonstrated excellent discriminatory power of salivary QA and its related metabolite ratios (Fig. 2). QA exhibited an area under curve (AUC) of 0.857, with a sensitivity of 87.1% and specificity of 75.8% at the optimal cut-off value (Table 2). The QA/Kyn ratio yielded a similar AUC of 0.855 but demonstrated improved specificity (78.8%) while maintaining comparable sensitivity. KA also showed good discriminatory ability (AUC 0.757), although with slightly lower specificity. These findings suggest that QA and its enzymatic ratio represent robust, non-invasive salivary biomarkers for distinguishing SjD from HCs.

**Table 2:** Discriminatory performance of salivary biomarkers between Sjögren’s disease and healthy controls.

	AUC	*p* value	Sensitivity	Specificity	Cut-off values
QA	0.857	< 0.001	0.871	0.758	2.1 nmol/mL
QA/Kyn ratio	0.855	< 0.001	0.839	0.788	11.6
KA	0.757	< 0.001	0.848	0.742	24.4 ng/mL

AUC: area under curve, QA: quinolinic acid, Kyn: kynurenine, KA: kynurenic acid.

### Correlations between salivary metabolites and clinical parameters

**Fig. 3:**
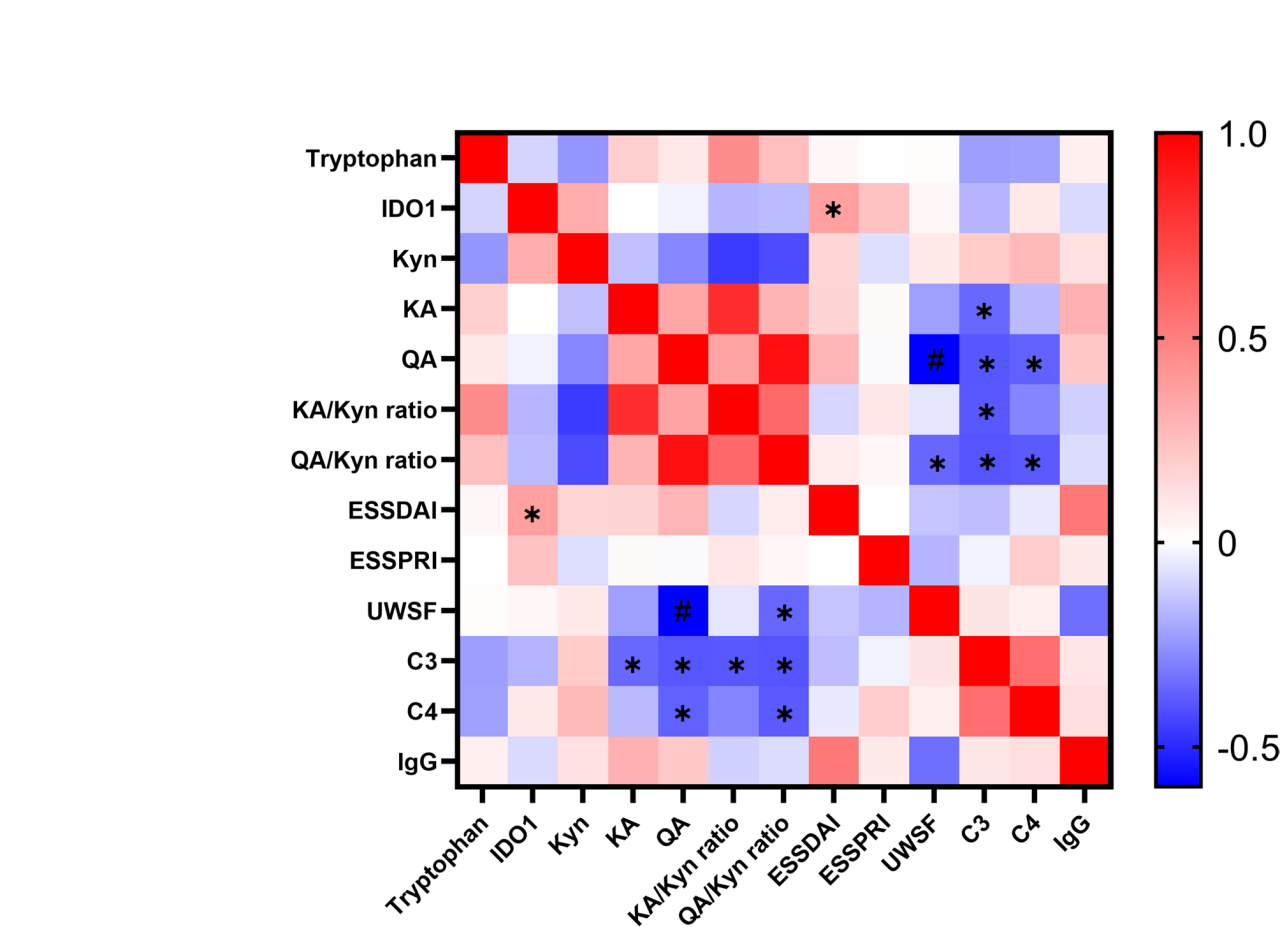
Correlations between salivary markers and clinical parameters of Sjögren’s disease. All statistical analyses were performed using the Spearman’s rank correlation coefficient test. IDO1: indoleamine-2,3-dioxygenase 1, Kyn: kynurenine, KA: kynurenic acid, QA: quinolinic acid, ESSDAI: EULAR Sjogren’s syndrome disease activity index, ESSPRI: EULAR Sjogren’s syndrome patient-reported index, UWSF: unstimulated whole salivary flow, IgG: immunoglobulin G, *p < 0.05, #: *p* < 0.0001.

Correlation analyses were performed to assess the relationships between salivary KP metabolites and clinical parameters (Fig. 3). QA levels exhibited strong inverse correlations with UWSFR (*p* < 0.001), as well as with serum C3 (*r* = -0.396, *p* = 0.017) and C4 levels (*r* = -0.372, *p* = 0.025). To further examine potential organ-specific associations, we performed an exploratory comparison of salivary QA levels according to the presence of EGMs. As summarized in Supplementary Table 2, there were no significant differences in QA levels between patients with and without EGMs. This finding suggests that salivary QA mainly reflects glandular dysfunction rather than systemic organ involvement. The QA/Kyn ratio, reflecting KMO activity, demonstrated similar trends, although the statistical significance was slightly lower than that of QA. Additionally, serum IDO1 levels were positively correlated with ESSDAI scores, indicating a potential link with systemic disease activity. KA and the KA/Kyn ratio showed moderate inverse correlations with serum C3 levels but were not significantly associated with measures of glandular dryness. We further examined whether salivary metabolite levels were associated with labial salivary gland histopathology. As summarized in Supplementary Table 3, none of the measured biomarkers showed statistically significant correlations with the focus score. This result suggests that salivary KP alterations are more closely related to functional rather than structural glandular changes. Collectively, these findings underscore that salivary QA levels are closely associated with both local glandular dysfunction and serologic markers of systemic immune activation in SjD.

### QA expression and clinical subgroup analysis

**Fig. 4:**
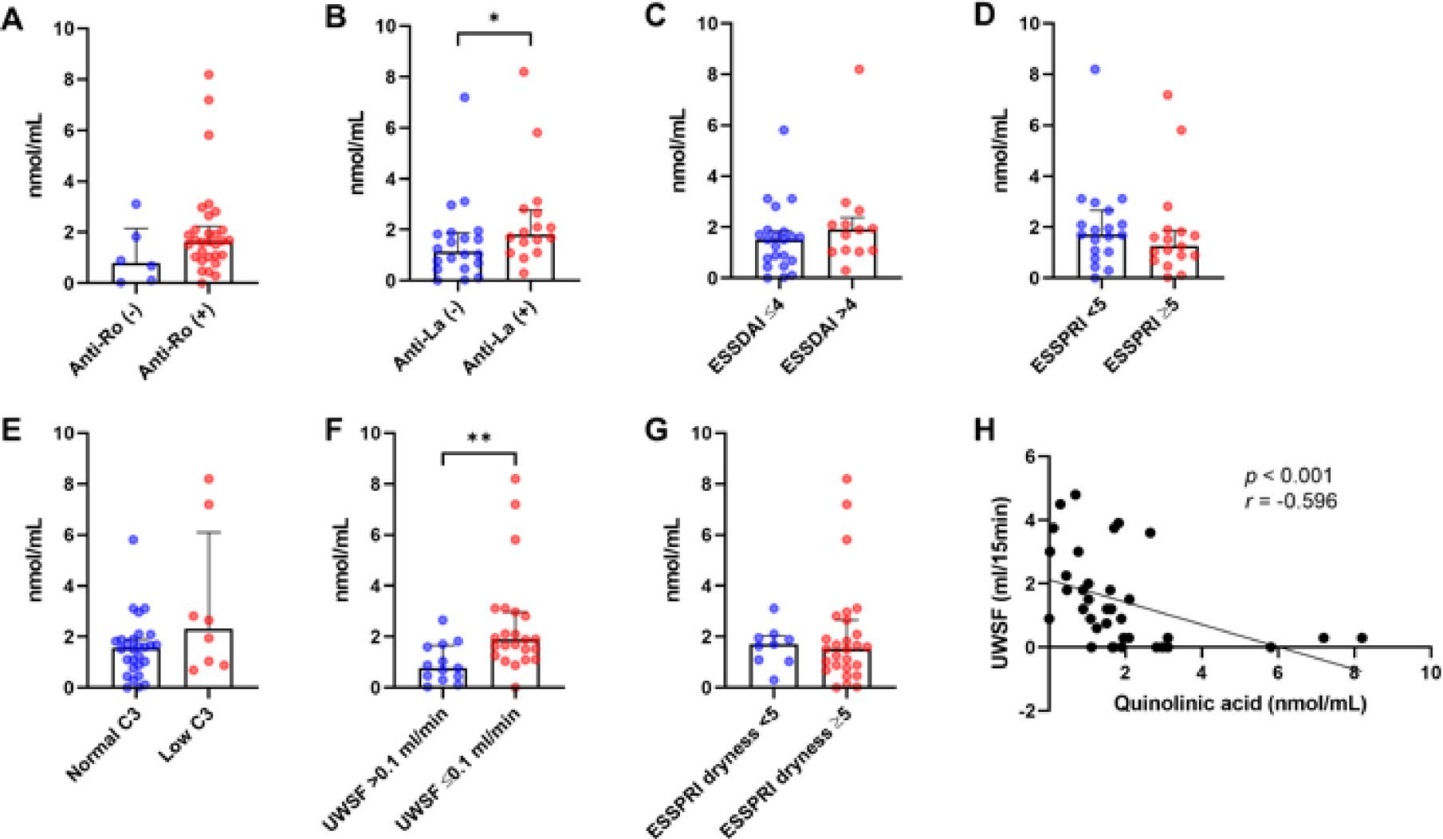
Expression levels of quinolinic acid in saliva from patients with Sjögren’s disease depending on clinical parameters (**A**–**G**). Correlations between UWSF and salivary quinolinic acid (**H**). Bars indicate the median and interquartile ranges. All statistical analyses were performed using the Mann-Whitney U test (**A**–**G**) and the Spearman’s rank correlation coefficient test (**H**). ESSDAI: EULAR Sjogren’s syndrome disease activity index, ESSPRI: EULAR Sjogren’s syndrome patient-reported index. UWSF: unstimulated whole salivary flow, *p < 0.05, **p < 0.01.

Given the close associations between salivary QA levels and key clinical parameters in SjD, we conducted additional subgroup analyses based on clinical features (Fig. 4). There was no significant difference in salivary QA levels based on anti-Ro antibody positivity (Fig. 4A). However, patients positive for anti-La antibodies exhibited significantly higher QA levels compared to those who were negative (Fig. 4B). QA levels did not differ significantly according to ESSDAI or overall ESSPRI scores, or serum C3 levels (Fig. 4C–E). In contrast, patients with UWSFR ≤ 0.1 mL/min had significantly lower salivary QA levels than those with preserved salivary flow (Fig. 4F; *p* < 0.01). No significant difference was observed based on the dryness domain score of the ESSPRI (Fig. 4G). A strong inverse correlation between salivary QA levels and UWSFR was again confirmed in scatterplot analysis (Fig. 4H; *r* = -0.596, *p* < 0.001), consistent with the findings in Fig. 3. These findings further support the potential of salivary QA as a sensitive marker, particularly for assessing glandular dysfunction in SjD.

## Discussion

This study provides comprehensive evidence that the salivary KP is significantly dysregulated in patients with SjD. We observed altered concentrations of multiple KP metabolites, including a marked reduction in salivary QA and increased levels of KA, alongside elevated IDO1 protein levels and shifts in downstream enzymatic ratios (KA/Kyn and QA/Kyn). These metabolic shifts were found to correlate with both glandular function, as assessed by UWSFR, and systemic immunologic features such as complement levels and disease activity indices. Notably, salivary QA and the QA/Kyn ratio demonstrated excellent discriminatory performance in distinguishing SjD from HCs, with AUC values exceeding 0.85. Subgroup analysis further underscored the relevance of QA as a biomarker of glandular dysfunction, with significantly lower levels observed in patients with reduced salivary flow. Taken together, our findings support the potential of salivary QA as a robust and non-invasive biomarker for indicating impaired salivary gland function in SjD.

Our results demonstrate a consistent pattern of KP dysregulation, with increased flux through the KAT branch and diminished activity of the KMO branch. This was evident from the elevated KA/Kyn ratio and the markedly decreased QA/Kyn ratio in patients with SjD compared to HCs (Fig. 1H and I). These findings suggest a metabolic shift away from the QA-producing arm of the pathway, which may reflect suppressed KMO activity. This pattern is consistent with previous studies in autoimmune thyroiditis and SLE, where similar imbalances in KP enzyme activity have been reported^20,21^. The discriminatory performance of salivary QA was particularly notable. In our ROC analysis, QA achieved an AUC of 0.857, with a sensitivity of 87.1% and specificity of 75.8%, while the QA/Kyn ratio demonstrated comparable performance with improved specificity (Fig. 2; Table 2). These values are similar to or better than other known salivary or tissue-based biomarkers, such as Kyn identified through salivary gland metabolomics^13^. Importantly, these results were obtained using non-invasive whole saliva samples, supporting the feasibility of implementing salivary QA measurement as a diagnostic tool in routine clinical practice. The clinical relevance of reduced QA was further supported by correlation analyses. QA levels were strongly and inversely correlated with UWSFR and serum complement levels (C3 and C4), suggesting that lower QA may reflect both local glandular dysfunction and systemic immune activation. Moreover, QA levels were significantly lower in patients with severely impaired salivary flow (UWSFR ≤ 0.1 mL/min), but not associated with subjective dryness scores or systemic disease activity indices (ESSDAI and ESSPRI). These findings indicate that QA may serve as a more objective and reliable indicator of glandular integrity than patient-reported outcomes.

Mechanistically, the observed reduction in QA levels could be due to impaired KMO activity, as supported by the decreased QA/Kyn ratio^22^. KMO is expressed in antigen-presenting cells and activated T cells, where it modulates Th1 and Th17 responses and contributes to the regulation of inflammatory tone^23^. These findings suggest that the dysregulated KP profile observed in SjD, characterized by elevated KA and decreased QA, may reflect altered immunometabolic signaling within glandular immune microenvironments. Although immunophenotyping data were not available in the current cohort, it is plausible that changes in local T cell and B cell activation, as well as antigen-presenting cell function, influence the observed metabolic pattern. Considering these, the observed reduction in QA levels and KMO activity may reflect a compensatory response to the chronic inflammatory environment of SjD, aimed at limiting excessive immune activation. However, despite this potential counter-regulatory role, QA is also known to exert proinflammatory and neurotoxic effects^24^. The persistent elevation of inflammatory mediators and local immune cell activation in the salivary microenvironment may contribute to cumulative tissue damage, ultimately resulting in a net decrease in salivary QA due to epithelial destruction and impaired enzymatic conversion in SjD. This is supported by the strong inverse correlation between QA levels and UWSFR, suggesting that salivary QA may serve as a marker of accumulated glandular injury in SjD. In contrast, KA levels were significantly elevated in SjD patients but showed weaker correlations with clinical parameters. KA is generally considered an anti-inflammatory and neuroprotective metabolite, acting via antagonism of N-methyl-D-aspartate receptors and activation of G protein-coupled receptor 35^25,26^. Its accumulation in saliva may reflect a compensatory mechanism or merely a metabolic consequence of altered KAT activity. The precise functional implications of elevated KA in the salivary environment remain unclear and warrant further investigation. Future studies integrating salivary metabolite profiling with flow-cytometric characterization of circulating and glandular immune-cell subsets would help clarify how immune activation shapes local tryptophan metabolism in SjD.

Our findings also confirmed increased IDO1 expression in SjD saliva, though the Kyn/Trp ratio, a conventional surrogate marker of IDO1 activity^27^, did not differ significantly between groups (Fig. 1F and G). This discrepancy may result from variable substrate availability, compensatory metabolic adjustments, or limitations in using static ratios as proxies for enzyme activity. Nonetheless, IDO1 protein levels were positively correlated with systemic disease activity (ESSDAI), suggesting a role in broader immune regulation. Although IDO1 is classically induced by IFN-γ, no significant difference in salivary IFN-γ was observed between SjD patients and HCs (Fig. 1A). This apparent discrepancy may reflect that IDO1 can also be upregulated by type I interferons and tumor necrosis factor-α, which are elevated in SjD independent of IFN-γ^3,28^. In addition, local cytokine activity within glandular infiltrates may not be fully captured by soluble salivary levels because of rapid turnover and dilutional effects. These observations suggest that salivary IDO1-related KP activation represents the cumulative influence of multiple cytokine pathways rather than direct proportionality to IFN-γ concentrations^28^.

Previous studies have reported dysregulation of the KP in various connective tissue diseases (CTD), including SLE and RA^8–10^. For instance, increased serum Kyn and elevated Kyn/Trp ratios have been associated with fatigue and systemic inflammation in SLE^8,9^ and with disease activity in RA^10^. However, the pattern observed in the present study, characterized by decreased salivary QA and a reduced QA/Kyn ratio, appears distinct from those systemic profiles and may reflect local metabolic impairment specific to the salivary gland microenvironment in SjD. Nonetheless, since only HCs were included for comparison, we acknowledge that our findings cannot definitively establish disease specificity. In addition, parallel analysis of KP metabolites in multiple biofluids could provide further insight into the tissue specificity of our findings. Previous studies have reported increased Kyn/Trp ratios in the serum of patients with SjD^11^ and SLE^8^, reflecting systemic immune activation. In contrast, the present study revealed a reduction in salivary QA and QA/Kyn ratios, which may represent a gland-specific metabolic signature associated with local epithelial dysfunction. Consistent with this interpretation, a targeted saliva metabolomics study by Piacenza Florezi et al. demonstrated that salivary metabolites closely mirror the glandular microenvironment in SjD^14^. Although we were unable to include serum or tear fluid analyses in the current cohort, these previous findings support the biological relevance of salivary QA as a potential indicator of glandular metabolic disturbance. Future studies incorporating other CTD cohorts and parallel biofluid assessments are warranted to validate the gland-specific relevance of salivary KP metabolites in SjD.

This study offers several strengths. We utilized saliva as a non-invasive, patient-friendly biospecimen that directly reflects local glandular conditions. Our approach included both quantitative metabolite measurements and enzymatic activity inference, enabling a comprehensive view of pathway dynamics. This study utilized data from a rigorously organized, nationwide prospective cohort, thereby enhancing the validity and robustness of the findings. Moreover, our data are consistent with prior reports from tissue, serum, and omics-based studies, reinforcing the biological plausibility and relevance of our results. However, several limitations should be acknowledged. The sample size was relatively modest. While the study utilized data from a nationwide cohort infrastructure, the enrolled subjects were all derived from a single-center population, potentially restricting the external validity of the findings. Longitudinal data are lacking, preventing assessment of whether salivary QA levels change in response to disease progression or treatment. In addition, we did not evaluate other KP metabolites such as 3-hydroxykynurenine or anthranilic acid^29^, which may provide additional insight into the metabolic landscape of SjD. Future studies incorporating broader metabolomic profiling and functional validation are warranted.

In conclusion, this study identifies salivary QA and its related metabolic ratios as promising non-invasive biomarkers for SjD. The strong discriminatory performance and close correlation with objective glandular dysfunction support its potential utility in detection and monitoring of the disease. These findings highlight the value of saliva as a biomarker medium and provide a foundation for future research into immunometabolic pathways in autoimmune exocrinopathies.

## Supplementary Information

Below is the link to the electronic supplementary material.

Supplementary Material 1

## Data Availability

The datasets generated during and/or analysed during the current study are available from the corresponding author on reasonable request.
