# Supplementary material for: Salivary kynurenine pathway metabolites as potential non-invasive markers of glandular dysfunction in Sjögren’s disease
Source: Sci Rep. 2025 Nov 18;15:40539. doi: 10.1038/s41598-025-24287-y (PMC12627724; doi:10.1038/s41598-025-24287-y)
Supplement: Supplementary file 1 — Supplementary Material 1 [file 41598_2025_24287_MOESM1_ESM.docx]

**Supplementary Materials**

**Supplementary Table 1.** Definitions of each type of extraglandular manifestations

| **Items** | **Definitions** |
| --- | --- |
| Articular involvement | A history or presence of morning stiffness over 30 minutes or any swollen or tender joints with the exclusion of degenerative arthritis |
| Raynaud’s phenomenon | The presence of at least two color changes in distal phalanges including pallor, cyanosis, and redness combined with sensory changes, such as pain and tingling |
| Lymphadenopathy | The presence of palpable lymph nodes over 1 cm |
| Lymphoma | A history of lymphoma confirmed by pathology |
| Liver involvement | The presence of autoimmune hepatitis or primary biliary cholangitis diagnosed clinically or pathologically with the exclusion of toxic hepatitis and transient elevation of liver enzymes |
| Splenomegaly | A palpable spleen below the left costal margin during full inspiration, or a splenic length ≥12 cm on imaging studies such as CT or ultrasound |
| Pulmonary involvement | A history or presence of autoimmune-mediated or interstitial lung disease |
| Cutaneous vasculitis | The presence of palpable purpura suggesting the extravasation of red blood cells into the dermis |
| Myositis | The presence of myositis diagnosed by elevated serum muscle enzymes, such as creatinine kinase and abnormal findings suggesting myositis in electromyography or biopsy |
| Peripheral neuropathy | Abnormal findings suggesting peripheral neuropathy in electroneurography |
| Central nervous system involvement | The presence of cranial or optic neuritis or cerebrovascular accident or epilepsy |
| Autoimmune thyroid disease | A history or presence of Graves’ disease or Hashimoto’s thyroiditis |
| Kidney involvement | The presence of interstitial nephritis combined with renal tubular acidosis or glomerulonephritis combined with proteinuria over 0.5 g/day |

**Supplementary Table 2**. Comparison of salivary quinolinic acid (QA) levels according to the presence of extraglandular manifestations (EGM)

|  | *n* (%) of EGM | Salivary QA with EGM, nmol/mL | Salivary QA without EGM, nmol/mL | *p* value |
| --- | --- | --- | --- | --- |
| Articular involvement | 15 (38.5) | 1.6 (1.0–1.9) | 1.7 (0.8–2.7) | 0.736 |
| Raynaud’s phenomenon | 5 (12.8) | 2.1 (0.1–3.1) | 1.6 (0.9–2.0) | 0.909 |
| Lymphadenopathy | 1 (2.6) | 1.9 | 1.6 (0.9–2.1) | 0.360 |
| Lymphoma | 0 (0) | N/A | N/A | N/A |
| Liver involvement | 1 (2.6) | 1.8 | 1.6 (0.9–2.1) | 0.312 |
| Splenomegaly | 0 (0) | N/A | N/A | N/A |
| Pulmonary involvement | 1 (2.6) | 1.7 | 1.6 (0.9–2.1) | 0.596 |
| Cutaneous vasculitis | 4 (10.3) | 1.5 (1.1–5.9) | 1.6 (0.8–2.1) | 0.546 |
| Myositis | 1 (2.6) | 1.9 | 1.6 (0.9–2.1) | 0.312 |
| Peripheral neuropathy | 3 (7.7) | 2.1 (1.9–2.1) | 1.5 (0.8–2.0) | 0.144 |
| Central nervous system involvement | 2 (5.1) | 1.5 (0.3–1.5) | 1.6 (0.9–2.1) | 0.782 |
| Autoimmune thyroid disease | 5 (12.8) | 0.9 (0.2–1.6) | 1.7 (1.0–2.7) | 0.078 |
| Kidney involvement | 1 (2.6) | 1.9 | 1.6 (0.9–2.1) | 0.312 |

Data are shown as *n* (%) or median (interquartile range). N/A: not applicable.

**Supplementary Table 3**. Correlation between salivary kynurenine pathway metabolites and labial salivary gland focus score in patients with Sjögren’s disease

| **Salivary biomarker** | **Spearman’s correlation coefficient** | ***p* value** |
| --- | --- | --- |
| Interferon-gamma | -0.109 | 0.656 |
| Tryptophan | 0.485 | 0.075 |
| IDO1 | 0.153 | 0.545 |
| Kynurenine | 0.103 | 0.684 |
| Kynurenic acid | -0.062 | 0.806 |
| Quinolinic acid | 0.114 | 0.651 |

IDO1: indoleamine-2,3-dioxygenase 1.
